# Hardy-type inequalities in fractional *h*-discrete calculus

**DOI:** 10.1186/s13660-018-1662-6

**Published:** 2018-04-04

**Authors:** Lars-Erik Persson, Ryskul Oinarov, Serikbol Shaimardan

**Affiliations:** 1 Luleå University of Technology, Luleå, Sweden; 2 UiT The Artic University of Norway, Narvik, Norway; 3 L. N. Gumilyev Eurasian National University, Astana, Kazakhstan

**Keywords:** 39A12, 49J05, 49K05, Inequality, Integral operator, *h*-calculus, *h*-integral, Discrete Fractional Calculus

## Abstract

The first power weighted version of Hardy’s inequality can be rewritten as

$$ \int _{0}^{\infty } \biggl( x^{\alpha -1} \int _{0}^{x} \frac{1}{t ^{\alpha }}f(t)\,dt \biggr) ^{p}\,dx\leq \biggl[ \frac{p}{p-\alpha -1} \biggr] ^{p} \int _{0}^{\infty }f^{p}(x)\,dx,\quad f\geq 0,p\geq 1, \alpha < p-1, $$

where the constant $C= [ \frac{p}{p-\alpha -1} ] ^{p}$ is sharp. This inequality holds in the reversed direction when $0\leq p<1$. In this paper we prove and discuss some discrete analogues of Hardy-type inequalities in fractional *h*-discrete calculus. Moreover, we prove that the corresponding constants are sharp.

## Introduction

The theory of fractional *h*-discrete calculus is a rapidly developing area of great interest both from a theoretical and applied point of view. Especially we refer to [1–8] and the references therein. Concerning applications in various fields of mathematics we refer to [9–16] and the references therein. Finally, we mention that *h*-discrete fractional calculus is also important in applied fields such as economics, engineering and physics (see, e.g. [17–22]).

Integral inequalities have always been of great importance for the development of many branches of mathematics and its applications. One typical such example is Hardy-type inequalities, which from the first discoveries of Hardy in the twentieth century now have been developed and applied in an almost unbelievable way, see, e.g., monographs [23] and [24] and the references therein. Let us just mention that in 1928 Hardy [25] proved the following inequality:

1.1 $$\begin{aligned} \int _{0}^{\infty } \biggl( x^{\alpha -1} \int _{0}^{x} \frac{1}{t ^{\alpha }}f(t)\,dt \biggr) ^{p}\,dx\leq \biggl( \frac{p}{p-\alpha -1} \biggr) ^{p} \int _{0}^{\infty }f^{p}(x)\,dx,\quad f\geq 0, \end{aligned}$$

for $1\leq p<\infty $ and $\alpha < p-1$ and where the constant $[ \frac{p}{p-\alpha -1} ] ^{p}$ is best possible. Inequality (1.1) is just a reformulation of the first power weighted generalization of Hardy’s original inequality, which is just (1.1) with $\alpha =0$ (so that $p>1$) (see [26] and [27]). Up to now there is no sharp discrete analogue of inequality (1.1). For example, the following two inequalities were claimed to hold by Bennett([28, p. 40–41]; see also [29, p. 407]):

$$ \sum _{n=1}^{\infty } \Biggl[ \frac{1}{n^{1-\alpha }}\sum_{k=0}^{n} \bigl[ k^{\alpha -1}-(k-1)^{\alpha -1} \bigr] a_{k} \Biggr] ^{p}\leq \biggl[ \frac{1-\alpha }{p-\alpha {p}-1} \biggr] ^{p}\sum _{n=1}^{\infty }{a}_{n}^{p},\quad a_{n}\geq 0, $$

and

$$ \sum _{n=1}^{\infty } \Biggl[ \frac{1}{\sum_{k=1}^{n}\frac{1}{k ^{-\alpha }}}\sum_{k=1}^{n}k^{-\alpha }a_{k} \Biggr] ^{p}\leq \biggl[ \frac{1-\alpha }{p-\alpha {p}-1} \biggr] ^{p}\sum_{n=1} ^{\infty }{a}_{n}^{p},\quad a_{n}\geq 0, $$

whenever $\alpha > 0$, $p > 1$, $\alpha {p}>1$. Both inequalities were proved independently by Gao [30, Corollary 3.1–3.2] (see also [31, Theorem 1.1] and [32, Theorem 6.1]) for $p\geq 1$ and some special cases of *α* (this means that there are still some regions of parameters with no proof of (1.1)). Moreover, in [33, Theorems 2.1 and 2.3] proved another sharp discrete analogue of inequality (1.1) in the following form:

$$ \sum _{n=-\infty }^{\infty } \Biggl[ \frac{1}{q^{n\lambda }} \sum_{k=0}^{n}q^{k\lambda }a_{k} \Biggr] ^{p}\leq \frac{1}{ ( 1-q ^{\lambda } ) ^{p}}\sum _{n=-\infty }^{\infty }{a}_{n}^{p}, \quad a_{n}\geq 0, $$

and

$$ \sum _{n=1}^{\infty } \Biggl[ \frac{1}{q^{n\lambda }}\sum_{k=0}^{n}q^{k\lambda }a_{k} \Biggr] ^{p}\leq \frac{1}{ ( 1-q^{ \lambda } ) ^{p}}\sum _{n=1}^{\infty }{a}_{n}^{p},\quad a _{n}\geq 0, $$

for $0 < q < 1$, $p\geq 1$ and $\alpha < 1-1/p$, where $\lambda :=1-1/p- \alpha $.

The main aim of this paper is to establish the *h*-analogue of the classical Hardy-type inequality (1.1) in fractional *h*-discrete calculus with sharp constants which is another discrete analogue of inequality (1.1).

The paper is organized as follows: In order not to disturb our discussions later on some preliminaries are presented in Sect. 2. The main results (see Theorem 3.1 and Theorem 3.2) with the detailed proofs can be found in Sect. 3.

## Preliminaries

We state the some preliminary results of the *h*-discrete fractional calculus which will be used throughout this paper.

Let $h>0$ and $\mathbb{T}_{a} :=\{a, a+h, a+2h,\ldots \}$, $\forall a \in \mathbb{R}$.

### Definition 2.1

(see [34])

Let $f:\mathbb{T}_{a}\rightarrow \mathbb{R}$. Then the *h*-derivative of the function $f=f(t)$ is defined by

2.1 $$\begin{aligned} D_{h}f(t):=\frac{f(\delta_{h}(t))-f(t)}{h},\quad t\in \mathbb{T}_{a}, \end{aligned}$$

where $\delta_{h}(t):=t+h$.

Let $fg : \mathbb{T}_{a} \rightarrow \mathbb{R}$. Then the product rule for *h*-differentiation reads (see [34])

2.2 $$\begin{aligned} D_{h} \bigl( f(x)g(x) \bigr) :=f(x)D_{h}g(x)+g(x+h)D_{h}f(x). \end{aligned}$$

The chain rule formula that we will use in this paper is

2.3 $$\begin{aligned} D_{h} \bigl[ x^{\gamma }(t) \bigr] :=\gamma \int _{0}^{1} \bigl[ zx\bigl( \delta_{h}(t) \bigr)+(1-z)x(t) \bigr] ^{\gamma -1}\,dzD_{h}x(t), \quad \gamma \in \mathbb{R}, \end{aligned}$$

which is a simple consequence of Keller’s chain rule [35, Theorem 1.90]. The integration by parts formula is given by (see [34]) the following.

### Definition 2.2

Let $f:\mathbb{T}_{a}\rightarrow \mathbb{R}$. Then the *h*-integral (*h*-difference sum) is given by

$$ \int _{a}^{b}f(x)\,d_{h}x:=\sum_{k=a/h}^{b/h-1}f(kh)h= \sum _{k=0}^{\frac{b-a}{h}-1}f(a+kh)h, $$

for $a,b\in \mathbb{T}_{a}, b>a$.

### Definition 2.3

We say that a function $g : \mathbb{T}_{a} \longrightarrow \mathbb{R}$, is nonincreasing (respectively, nondecreasing) on $\mathbb{T}_{a}$ if and only if $D_{h}g(t) \leq 0$ (respectively, $D_{h}g(t) \geq 0$) whenever $x\in \mathbb{T}_{a}$.

Let $D_{h}F(x) = f(x)$. Then $F(x)$ is called a *h*-antiderivative of $f(x)$ and is denoted by $\int f(x)\,d_{h}x$. If $F(x)$ is a *h*-antiderivative of $f(x)$, for $a,b\in \mathbb{T}_{a}, b>a$ we have (see [36])

2.4 $$\begin{aligned} \int _{a}^{b}f(x)\,d_{h}x:=F(b)-F(a). \end{aligned}$$

### Definition 2.4

(see [34])

Let $t,\alpha \in \mathbb{R}$. Then the *h*-fractional function $t_{h}^{(\alpha )}$ is defined by

$$ t_{h}^{(\alpha )}:=h^{\alpha }\frac{\Gamma (\frac{t}{h}+1)}{\Gamma ( \frac{t}{h}+1-\alpha )}, $$

where Γ is Euler gamma function, $\frac{t}{h}\notin \{-1, -2, -3, \ldots \}$ and we use the convention that division at a pole yields zero. Note that

$$ \lim _{h\rightarrow 0}t^{(\alpha )}_{h}=t^{\alpha }. $$

Hence, by (2.1) we find that

2.5 $$\begin{aligned} t_{h}^{(\alpha -1)}=\frac{1}{\alpha }D_{h} \bigl[ t_{h}^{(\alpha )} \bigr] . \end{aligned}$$

### Definition 2.5

The function $f:(0, \infty )\rightarrow \mathbb{R}$ is said to be log-convex if $f(ux+(1-u)y)\leq f^{u}(x)f^{1-u}(y)$ holds for all $x,y\in (0, \infty )$ and $0< u<1$.

Next, we will derive some properties of the *h*-fractional function, which we need for the proofs of the main results, but which are also of independent interest.

### Proposition 2.6

*Let* $t\in \mathbb{T}_{0}$. *Then*, *for* $\alpha ,\beta \in \mathbb{R}$,

2.6 $$\begin{aligned} t_{h}^{(\alpha +\beta )} =&t_{h}^{(\alpha )}{(t- \alpha h)}_{h}^{( \beta )}, \end{aligned}$$

2.7 $$\begin{aligned} t_{h}^{(p\alpha )}\leq \bigl[ t_{h}^{(\alpha )} \bigr] ^{p}\leq \bigl(t+ \alpha (p-1)h\bigr)_{h}^{(p\alpha )}, \ \end{aligned}$$

*for* $1\leq p<\infty $, *and*

2.8 $$\begin{aligned} \bigl[ t_{h}^{(\alpha )} \bigr] ^{p}\leq t_{h}^{(p\alpha )}, \end{aligned}$$

*for* $0< p<1$.

### Proof

By using Definition 2.4 we get

$$\begin{aligned} t_{h}^{(\alpha +\beta )} =&h^{\alpha +\beta }\frac{\Gamma ( \frac{t}{h}+1)}{\Gamma (\frac{t}{h}+1-\alpha -\beta )} \\ =&h^{\alpha }\frac{\Gamma (\frac{t}{h}+1)}{\Gamma (\frac{t}{h}+1- \alpha )}h^{\beta } \frac{\Gamma (\frac{t}{h}+1-\alpha )}{\Gamma ( \frac{t}{h}+1-\alpha -\beta )}=t_{h}^{(\alpha )}{(t- \alpha h)}_{h} ^{(\beta )}, \end{aligned}$$

i.e. (2.6) holds for $\alpha ,\beta \in \mathbb{R}$.

It is well known that the gamma function is log-convex (see, e.g., [37], p. 21). Hence,

$$\begin{aligned} \bigl[ t_{h}^{(\alpha )} \bigr] ^{p} =&h^{p\alpha } \biggl[ \frac{\Gamma ( \frac{t}{h}+1)}{\Gamma (\frac{t}{h}+1-\alpha )} \biggr] ^{p} \\ =&h^{p\alpha } \biggl[ \frac{\Gamma (\frac{1}{p}(\frac{t}{h}+1+\alpha (p-1))+(1- \frac{1}{p})(\frac{t}{h}+1-\alpha ))}{\Gamma (\frac{t}{h}+1-\alpha )} \biggr] ^{p} \\ \leq &h^{p\alpha } \biggl[ \frac{\Gamma^{\frac{1}{p}}(\frac{1}{h}+1+ \alpha (p-1))\Gamma^{1-\frac{1}{p}}(\frac{t}{h}+1-\alpha )}{\Gamma ( \frac{t}{h}+1-\alpha )} \biggr] ^{p} \\ =&h^{p\alpha }\frac{\Gamma (\frac{t}{h}+1+\alpha (p-1))}{\Gamma ( \frac{t}{h}+1-\alpha )}=\bigl(t+\alpha (p-1)h\bigr)_{h}^{(p\alpha )} \end{aligned}$$

and

$$\begin{aligned} \bigl[ t_{h}^{(\alpha )} \bigr] ^{p} =&h^{p\alpha } \biggl[ \frac{\Gamma ( \frac{t}{h}+1)}{\Gamma (\frac{t}{h}+1-\alpha )} \biggr] ^{p} \\ =&h^{p\alpha } \biggl[ \frac{\Gamma (\frac{t}{h}+1)}{\Gamma ((1- \frac{1}{p})(\frac{t}{h}+1)+\frac{1}{p}(\frac{t}{h}+1-p\alpha ))} \biggr] ^{p} \\ \geq &h^{p\alpha } \biggl[ \frac{\Gamma (\frac{t}{h}+1)}{ \Gamma^{1-\frac{1}{p}}(\frac{t}{h}+1)\Gamma^{\frac{1}{p}}(\frac{t}{h}+1-p \alpha )} \biggr] ^{p} \\ =&h^{p\alpha }\frac{\Gamma (\frac{t}{h}+1)}{\Gamma (\frac{t}{h}+1-p \alpha )}=t_{h}^{(p\alpha )}, \end{aligned}$$

so we have proved that (2.7) holds wherever $1\leq p< \infty $. Moreover, for $0< p<1$,

$$\begin{aligned} t_{h}^{(p\alpha )} =&h^{p\alpha }\frac{\Gamma (\frac{t}{h}+1)}{\Gamma (\frac{t}{h}+1-p\alpha )} \\ =&h^{p\alpha }\frac{\Gamma (\frac{t}{h}+1)}{\Gamma ((1-p)( \frac{t}{h}+1)+p(\frac{t}{h}+1-\alpha ))} \\ \geq &h^{p\alpha }\frac{\Gamma (\frac{t}{h}+1)}{\Gamma^{(1-p)}( \frac{t}{h}+1)\Gamma^{p}(\frac{t}{h}+1-\alpha )} \\ =& \biggl[ h^{\alpha }\frac{\Gamma (\frac{t}{h}+1)}{\Gamma (\frac{t}{h}+1- \alpha )} \biggr] ^{p}= \bigl[ t_{h}^{(\alpha )} \bigr] ^{p}, \end{aligned}$$

so we conclude that (2.8) holds for $0< p<1$. The proof is complete. □

## Main results

Our *h*-integral analogue of inequality (1.1) reads as follows.

### Theorem 3.1

*Let* $\alpha <\frac{p-1}{p}$ *and* $1\leq p<\infty $. *Then the inequality*

3.1 $$\begin{aligned} \int _{0}^{\infty } \biggl( x^{(\alpha -1)}_{h} \int _{0}^{ \delta_{h}(x)}\frac{f(t)\,d_{h}t}{t_{h}^{(\alpha )}} \biggr) ^{p}\,d_{h}x \leq \biggl( \frac{p}{p-\alpha p-1} \biggr) ^{p} \int _{0}^{\infty }f^{p}(x)\,d_{h}x, \quad f\geq 0, \end{aligned}$$

*holds*. *Moreover*, *the constant* $[ \frac{p}{p-\alpha p-1} ] ^{p}$ *is the best possible in* (3.1).

Our second main result is the following *h*-integral analogue of the reversed form of (1.1) for $0< p<1$.

### Theorem 3.2

*Let* $\alpha <\frac{p-1}{p}$ *and* $0 < p < 1$. *Then the inequality*

3.2 $$\begin{aligned} \int _{0}^{\infty }f^{p}(x)\,d_{h}x \leq \biggl( \frac{p-p\alpha -1}{p} \biggr) ^{p} \int _{0}^{\infty } \biggl( x^{(\alpha -1)}_{h} \int _{0} ^{\delta_{h}(x)}\frac{f(t)\,d_{h}t}{t_{h}^{(\alpha )}} \biggr) ^{p}\,d_{h}x, \quad f\geq 0, \end{aligned}$$

*holds*. *Moreover*, *the constant* $[ \frac{p-p\alpha -1}{p} ] ^{p}$ *is the best possible in* (3.2).

To prove Theorem 3.1 we need the following lemma, which is of independent interest.

### Lemma 3.3

*Let* $\alpha <\frac{p-1}{p}$, $p>1$ *and* $\frac{1}{p}+\frac{1}{p'}=1$. *Then the function*

$$ \phi (x):= \biggl[ \biggl( x-\biggl(\alpha +\frac{1}{p}\biggr)h \biggr) _{h}^{(- \frac{1}{p})} \biggr] ^{\frac{1}{p'}} \biggl[ \biggl( x-\biggl( \alpha - \frac{1}{p'}\biggr)h \biggr) _{h}^{(\frac{1}{p'})} \biggr] ^{\frac{1}{p}},\quad x\in \mathbb{T}_{0}, $$

*is nonincreasing on* $\mathbb{T}_{0}$.

### Proof

Let $\alpha <\frac{p-1}{p}$ and $1\leq p<\infty $. Since $\Gamma (x)>0$ for $x>0$, and using Definition 2.4, we have

$$\begin{aligned} \biggl( x-\biggl(\alpha +\frac{1}{p}\biggr)h \biggr) _{h}^{(-\frac{1}{p})} =& h^{- \frac{1}{p}} \frac{\Gamma ( \frac{x}{h}+\frac{1}{p'}-\alpha ) }{ \Gamma ( \frac{x}{h}+\frac{1}{p}+\frac{1}{p'}-\alpha ) }>0 \end{aligned}$$

and

$$\begin{aligned} \biggl( x-\biggl(\alpha -\frac{1}{p'}\biggr)h \biggr) _{h}^{(\frac{1}{p'})} =& h^{ \frac{1}{p'}} \frac{\Gamma ( \frac{x}{h}+1+\frac{1}{p'}-\alpha ) }{ \Gamma ( \frac{x}{h}+1-\alpha ) }>0. \end{aligned}$$

Denote $\xi (x):= ( x-(\alpha +\frac{1}{p})h ) _{h}^{(- \frac{1}{p})}$ and $\eta (x):= ( x-(\alpha -\frac{1}{p'})h ) _{h}^{(\frac{1}{p'})}$. Then by using (2.5) we find that

3.3 $$\begin{aligned} D_{h}\eta (x)=\frac{ ( x-(\alpha -\frac{1}{p'})h ) _{h}^{(- \frac{1}{p})}}{p'}\geq 0 \end{aligned}$$

and

3.4 $$\begin{aligned} D_{h}\xi (x)=-\frac{ ( x-(\alpha +\frac{1}{p})h ) _{h}^{(- \frac{1}{p}-1)}}{p}\leq 0, \end{aligned}$$

From (2.3), (2.6), (3.3) and (3.4) it follows that

3.5 $$\begin{aligned} D_{h} \bigl[ \xi (x) \bigr] ^{\frac{1}{p'}} =& \frac{1}{p'} \int _{0}^{1} \bigl[ z\xi (x+h)+(1-z)\xi (x) \bigr] ^{-\frac{1}{p}}\,dz D_{h} \xi (x) \\ \leq &- \bigl[ \xi (x) \bigr] ^{-\frac{1}{p}} \frac{ ( x-(\alpha + \frac{1}{p})h ) _{h}^{(-\frac{1}{p}-1)}}{pp'} \\ \leq &- \bigl[ \xi (x) \bigr] ^{\frac{1}{p'}} \frac{ ( x-\alpha {h} ) _{h}^{(-1)}}{pp'} \end{aligned}$$

and

3.6 $$\begin{aligned} D_{h} \bigl[ \eta (x) \bigr] ^{\frac{1}{p}} =& \frac{1}{p} \int _{0}^{1} \bigl[ z\eta (x+h)+z\eta (x) \bigr] ^{-\frac{1}{p'}}\,dz D_{h} \eta (x) \\ \leq & \bigl[ \eta (x) \bigr] ^{-\frac{1}{p'}}\frac{ ( x-(\alpha - \frac{1}{p'})h ) _{h}^{(-\frac{1}{p})}}{pp'}. \end{aligned}$$

By using the fact that $( x+h-\alpha h ) _{h}^{(1)} ( x- \alpha h ) _{h}^{(-1)}=1$, $\eta (x+h)\geq \eta (x)$,

$$ \eta (x) \biggl[ \biggl( x-\biggl(\alpha -\frac{1}{p'}\biggr)h \biggr) _{h}^{(- \frac{1}{p})} \biggr] ^{-1}= ( x+h-\alpha h ) _{h}^{(1)}, $$

for $x\in \mathbb{T}_{0}$ and (2.2), (3.3), (3.4), (3.5) and (3.6) we obtain

$$\begin{aligned} D_{h} \bigl( \phi (x) \bigr) &= \bigl[ \xi (x) \bigr] ^{\frac{1}{p'}} D _{h} \bigl[ \eta (x) \bigr] ^{\frac{1}{p}} + \bigl[ \eta (x+h) \bigr] ^{1- \frac{1}{p'}} D_{h} \bigl[ \xi (x) \bigr] ^{\frac{1}{p'}} \\ &\leq \frac{ [ \xi (x) ] ^{\frac{1}{p'}} [ \eta (x) ] ^{-\frac{1}{p'}}}{pp'} \biggl[ \biggl( x-\biggl(\alpha -\frac{1}{p'} \biggr)h \biggr) _{h}^{(-\frac{1}{p})} -\eta (x) ( x-\alpha {h} ) _{h}^{(-1)} \biggr] \\ &=\frac{ [ \xi (x) ] ^{\frac{1}{p'}} [ \eta (x) ] ^{- \frac{1}{p'}}}{pp'} \biggl( x-\biggl(\alpha -\frac{1}{p'}\biggr)h \biggr) _{h}^{(- \frac{1}{p})} \bigl[ 1- ( x+h-\alpha h ) _{h}^{(1)} ( x-\alpha h ) _{h}^{(-1)} \bigr] \\ &\leq 0. \end{aligned}$$

Hence, we have proved that the function $\phi (x)$ is nonincreasing on $\mathbb{T}_{0}$ (see Definition 2.4) so the proof is complete. □

### Proof of Theorem 3.1

Let $p > 1$. By using Lemma 3.3 and (2.6) in Proposition 2.6 we have

3.7 $$\begin{aligned} x^{(\alpha -1)}_{h} &= \bigl[ x^{(\alpha -1)}_{h} \bigr] ^{\frac{1}{p'}} \bigl[ x^{(\alpha -1)}_{h} \bigr] ^{\frac{1}{p}} \\ &= \biggl[ x^{(\alpha -\frac{1}{p'})}_{h} \biggl( x-\biggl(\alpha - \frac{1}{p'}\biggr)h \biggr) _{h}^{(-\frac{1}{p})} \biggr] ^{\frac{1}{p'}} \biggl[ x^{(\alpha - \frac{1}{p'}-1)}_{h} \biggl( x-\biggl(\alpha -\frac{1}{p'}-1\biggr)h \biggr) _{h}^{( \frac{1}{p'})} \biggr] ^{\frac{1}{p}} \\ &= \bigl[ x^{(\alpha -\frac{1}{p'})}_{h} \bigr] ^{\frac{1}{p'}} \bigl[ x ^{(\alpha -\frac{1}{p'}-1)}_{h} \bigr] ^{\frac{1}{p}} \\ &\quad {}\times \biggl[ \biggl( x+h-\biggl(\alpha +\frac{1}{p}\biggr)h \biggr) _{h}^{(- \frac{1}{p})} \biggr] ^{\frac{1}{p'}} \biggl[ \biggl( x+h- \biggl(\alpha - \frac{1}{p'}\biggr)h \biggr) _{h}^{(\frac{1}{p'})} \biggr] ^{\frac{1}{p}} \\ &= \bigl[ x^{(\alpha -\frac{1}{p'})}_{h} \bigr] ^{\frac{1}{p'}} \bigl[ x ^{(\alpha -\frac{1}{p'}-1)}_{h} \bigr] ^{\frac{1}{p}}\phi (x+h) \\ &\leq \bigl[ x^{(\alpha -\frac{1}{p'})}_{h} \bigr] ^{\frac{1}{p'}} \bigl[ x^{(\alpha -\frac{1}{p'}-1)}_{h} \bigr] ^{\frac{1}{p}}\phi (t), \end{aligned}$$

for $t,x\in \mathbb{T}_{0}:t\leq x$. Moreover,

3.8 $$\begin{aligned} \frac{\phi (t)}{t_{h}^{(\alpha )}} =& \bigl[ (t-\alpha h)_{h}^{(-\alpha )} \bigr] ^{\frac{1}{p'}} \bigl[ (t-\alpha h)_{h}^{(-\alpha )} \bigr] ^{\frac{1}{p}}\phi (t) \\ =& \biggl[ (t-\alpha h)_{h}^{(-\alpha )}\biggl(t-\biggl(\alpha + \frac{1}{p}\biggr)h\biggr)_{h} ^{(-\frac{1}{p})} \biggr] ^{\frac{1}{p'}} \biggl[ (t-\alpha h)_{h}^{(- \alpha )}\biggl(t- \biggl(\alpha -\frac{1}{p'}\biggr) h\biggr)_{h}^{(\frac{1}{p'})} \biggr] ^{ \frac{1}{p}} \\ =& \biggl[ \biggl(t-\biggl(\alpha +\frac{1}{p}\biggr)h \biggr)_{h}^{(-\alpha -\frac{1}{p})} \biggr] ^{\frac{1}{p'}} \biggl[ \biggl(t- \biggl(\alpha -\frac{1}{p'}\biggr)h\biggr)_{h}^{(\frac{1}{p'}- \alpha )} \biggr] ^{\frac{1}{p}}. \end{aligned}$$

According to (3.7) and (3.8) we have

$$\begin{aligned} L(f) &:= \int _{0}^{\infty } \biggl( x^{(\alpha -1)}_{h} \int _{0}^{\delta_{h}(x)}\frac{1}{t_{h}^{(\alpha )}} f(t)\,d_{h}t \biggr) ^{p}\,d _{h}x \\ &\leq \int_{0}^{\infty } \biggl( \bigl[ x^{(\alpha -\frac{1}{p'})} _{h} \bigr] ^{\frac{1}{p'}} \bigl[ x^{(\alpha -\frac{1}{p'}-1)}_{h} \bigr] ^{\frac{1}{p}} \int _{0}^{\delta_{h}(x)} \biggl[ \biggl(t-\biggl(\alpha + \frac{1}{p}\biggr)h\biggr)_{h}^{(-\alpha -\frac{1}{p})} \biggr] ^{\frac{1}{p'}} \\ &\quad {}\times \biggl[ \biggl(t-\biggl(\alpha -\frac{1}{p'}\biggr) h \biggr)_{h}^{(\frac{1}{p'}- \alpha )} \biggr] ^{\frac{1}{p}}f(t)\,d_{h}t \biggr) ^{p}\,d_{h}x \\ &=\sum _{i=0}^{\infty }h^{1+p} \Biggl( \bigl[ (ih)^{(\alpha - \frac{1}{p'})}_{h} \bigr] ^{\frac{1}{p'}} \bigl[ (ih)^{(\alpha - \frac{1}{p'}-1)}_{h} \bigr] ^{\frac{1}{p}}\times \\ &\quad {}\times \sum _{k=0}^{i} \biggl[ \biggl(kh-\biggl(\alpha +\frac{1}{p}\biggr)h\biggr)_{h} ^{(-\alpha -\frac{1}{p})} \biggr] ^{\frac{1}{p'}} \biggl[ \biggl(kh-\biggl(\alpha - \frac{1}{p'}\biggr) h\biggr)_{h}^{(\frac{1}{p'}-\alpha )} \biggr] ^{\frac{1}{p}}f(kh) \Biggr) ^{p} \\ &=I^{p}(f). \end{aligned}$$

Let $\mathbb{N}_{0}=\mathbb{N}\cup \{0\}$, $g = \{g_{k}\}_{k=1}^{ \infty }\in {l}_{p'}(\mathbb{N}_{0})$, $g\geq 0$, and $\Vert g \Vert _{ {l}_{p'}}= 1$. Moreover, let $\theta (z)$ be Heaviside’s unit step function ($\theta (z) = 1$ for $z \geq 0$ and $\theta (z) = 0$ for $z < 0$). Then, based on the duality principle in ${l} _{p}(\mathbb{N}_{0})$ and the Hölder inequality, we find that

3.9 $$\begin{aligned} I(f) &=\sup_{\Vert g \Vert _{ {l}_{p'}}= 1}\sum _{i,k}h^{1+ \frac{1}{p}}g_{i} \theta (i-k) \bigl[ (ih)^{(\alpha -\frac{1}{p'})}_{h} \bigr] ^{\frac{1}{p'}} \bigl[ (ih)^{(\alpha -\frac{1}{p'}-1)}_{h} \bigr] ^{ \frac{1}{p}} \\ &\quad {}\times \biggl[ \biggl(kh-\biggl(\alpha +\frac{1}{p}\biggr)h \biggr)_{h}^{(-\alpha -\frac{1}{p})} \biggr] ^{\frac{1}{p'}} \biggl[ \biggl(kh- \biggl(\alpha -\frac{1}{p'}\biggr) h\biggr)_{h}^{( \frac{1}{p'}-\alpha )} \biggr] ^{\frac{1}{p}}f(kh) \\ &\leq \sup _{\Vert g \Vert _{ {l}_{p'}}= 1} \biggl( \sum _{i,k}hg _{i}^{p'}\theta (i-k) (ih)_{h}^{(\alpha -\frac{1}{p'})} \biggl(kh-\biggl(\alpha + \frac{1}{p}\biggr) h\biggr)_{h}^{(-\alpha -\frac{1}{p})} \biggr) ^{\frac{1}{p'}} \\ &\quad {}\times \biggl( \sum _{i,k}h^{2}\theta (i-k) (ih)_{h}^{(\alpha - \frac{1}{p'}-1)}\biggl(kh-\biggl(\alpha -\frac{1}{p'} \biggr) h\biggr)_{h}^{(\frac{1}{p'}- \alpha )}f^{p}(kh) \biggr) ^{\frac{1}{p}} \\ &=\sup _{\Vert g \Vert _{ {l}_{p'}}= 1}I_{1}^{p'}(g)I_{2}^{q}(f). \end{aligned}$$

By using Definition 2.3 and combining (2.4), (2.5) and (2.6) we can conclude that

3.10 $$\begin{aligned} I_{1}(g) =&\sum _{i=0}^{\infty }g_{i}^{p'}(ih)_{h}^{(\alpha - \frac{1}{p'})} \sum _{k=0}^{i}h\biggl(kh-\biggl(\alpha + \frac{1}{p}\biggr) h\biggr)_{h} ^{(-\alpha -\frac{1}{p})} \\ =&\sum _{i=0}^{\infty }g_{i}^{p'}(ih)_{h}^{(\alpha - \frac{1}{p'})} \int _{0}^{\delta_{h}(ih)}\biggl(x-\biggl(\alpha +\frac{1}{p} \biggr) h\biggr)_{h}^{(-\frac{1}{p}-\alpha )}\,d_{h}x \\ =&\frac{1}{\frac{1}{p'}-\alpha }\sum _{i=1}^{\infty }g_{i}^{p'}(ih)_{h} ^{(\alpha -\frac{1}{p'})} \int _{0}^{\delta_{h}(ih)}D_{h} \biggl[ \biggl(x-\biggl( \alpha +\frac{1}{p}\biggr) h\biggr)_{h}^{(\frac{1}{p'}-\alpha )} \biggr]\,d_{h}x \\ \leq &\frac{1}{\frac{1}{p'}-\alpha }\sum _{i=1}^{\infty }g_{i} ^{p'}(ih)_{h}^{(\alpha -\frac{1}{p'})}\biggl(ih-\biggl(\alpha - \frac{1}{p'}\biggr) h\biggr)_{h} ^{(\frac{1}{p'}-\alpha )} \\ =&\frac{1}{\frac{1}{p'}-\alpha }\Vert g \Vert ^{p'}_{ {l}_{p'}}= \frac{1}{ \frac{1}{p'}-\alpha }. \end{aligned}$$

Furthermore,

3.11 $$\begin{aligned} I_{2}(f) =&\sum _{i=0}^{\infty }h(ih)_{h}^{(\alpha - \frac{1}{p'}-1)} \sum _{k=0}^{i}hf^{p}(kh) \biggl(kh- \biggl(\alpha - \frac{1}{p'}\biggr)h\biggr)_{h}^{(\frac{1}{p'}-\alpha )} \\ =&\sum _{k=0}^{\infty }hf^{p}(kh) \biggl(kh- \biggl(\alpha -\frac{1}{p'}\biggr)h\biggr)_{h} ^{(\frac{1}{p'}-\alpha )} \sum _{i=k}^{\infty }h(ih)_{h}^{( \alpha -\frac{1}{p'}-1)} \\ =&\frac{1}{\alpha -\frac{1}{p'}} \int _{0}^{\infty }f^{p}(x) \biggl(x-\biggl( \alpha -\frac{1}{p'}\biggr)h\biggr)_{h}^{(\frac{1}{p'}-\alpha )} \int _{x} ^{\infty }D_{h} \bigl[ t_{h}^{(\alpha -\frac{1}{p'})} \bigr] \,d_{h}t\, d_{h}x \\ =&\frac{1}{\frac{1}{p'}-\alpha }\int_{0}^{\infty }f^{p}(x) \biggl(x-\biggl( \alpha-\frac{1}{p'}\biggr)h\biggr)_{h}^{(\frac{1}{p'}-\alpha )} x_{h}^{(\alpha - \frac{1}{p'})}\,d_{h}x \\ =&\frac{1}{\frac{1}{p'}-\alpha } \int _{0}^{\infty }f^{p}(x)\,d _{h}x. \end{aligned}$$

By combining (3.9), (3.10) and (3.11) we obtain

3.12 $$\begin{aligned} L(f)\leq \biggl( \frac{p}{p-p\alpha -1} \biggr) ^{p} \int _{0}^{ \infty }f^{p}(x)\,d_{h}x, \end{aligned}$$

i.e. (3.1) holds.

Finally, we will prove that the constant $[ \frac{p}{p-\alpha p-1} ] ^{p}$ is the best possible in inequality (3.1). Let $x,a\in \mathbb{T}_{0}$ be such that $a< x$, and consider the test function $f_{\beta }(t)=t_{h}^{(\beta )}\chi_{[a,\infty )}(t)$, $t>0$, for $\beta =-\frac{1}{p}-\varepsilon $.

Then from (2.4), (2.5) and (2.7) it follows that

$$\begin{aligned} \int _{0}^{\infty }f^{p}_{\beta }(t)\,d_{h}t =& \int _{a} ^{\infty } \bigl[ t_{h}^{(\beta )} \bigr] ^{p}\,d_{h}t\leq \int_{a} ^{\infty }\bigl(t+\beta (p-1)h\bigr)_{h}^{(\beta p)}\,d_{h}t \\ =&\frac{1}{p\beta +1} \int _{a}^{\infty }D_{h} \bigl[ \bigl(t+\beta (p-1)h\bigr)_{h} ^{(\beta p+1)} \bigr]\,d_{h}t \\ =&\frac{(a+\beta (p-1)h)_{h}^{(p\beta +1)}}{\vert p\beta +1 \vert }< \infty . \end{aligned}$$

Since

$$\begin{aligned} \biggl( \int _{0}^{\delta_{h}(x)} (t-h\alpha )_{h}^{(-\alpha )}f _{\beta }(t)\,d_{h}t \biggr) ^{p} =& \biggl( \int _{a}^{\delta_{h}(x)} (t-h\alpha )_{h}^{(-\alpha +\beta )}\,d_{h}t \biggr) ^{p} \\ =& \biggl( \frac{1}{1-\alpha +\beta } \int _{a}^{\delta_{h}(x)} D _{h} \bigl[ (t-h\alpha )_{h}^{(1-\alpha +\beta )} \bigr]\,d_{h}t \biggr) ^{p} \\ =& \biggl( \frac{(x+h-h\alpha )_{h}^{(1-\alpha +\beta )}}{1-\alpha + \beta } \biggl[ 1-\frac{(a-h\alpha )_{h}^{(1-\alpha +\beta )}}{(x+h-h \alpha )_{h}^{(1-\alpha +\beta )}} \biggr] \biggr) ^{p} \\ \geq & \biggl( \frac{(x+h-h\alpha )_{h}^{(1-\alpha +\beta )}}{1-\alpha +\beta } \biggr) ^{p} \biggl[ 1-p \frac{(a-h\alpha )_{h}^{(1-\alpha + \beta )}}{(x+h-h\alpha )_{h}^{(1-\alpha +\beta )}} \biggr] , \end{aligned}$$

we have

3.13 $$\begin{aligned} L(f_{\beta }) &\geq \biggl( \frac{1}{1-\alpha +\beta } \biggr) ^{p} \biggl[\int_{a}^{\infty } \bigl[ x_{h}^{(\alpha -1)}(x+h-h\alpha )_{h}^{(1-\alpha +\beta )} \bigr] ^{p}\,d_{h}x \\ &\quad {}-p(a-h\alpha )_{h}^{(1-\alpha +\beta )} \int _{a}^{ \infty }\frac{ [ x_{h}^{(\alpha -1)}(x+h-h\alpha )_{h}^{(1-\alpha +\beta )} ] ^{p}}{(x+h-h\alpha )_{h}^{(1-\alpha +\beta )}}\,d_{h}x \biggr] \\ &=\biggl( \frac{1}{1-\alpha +\beta } \biggr) ^{p} \biggl[\int_{0} ^{\infty }f_{\beta }^{p}(x)\,d_{h}x-p\int_{a}^{\infty }\frac{(a-h\alpha )_{h}^{(1-\alpha +\beta )} [ x_{h}^{(\beta )} ] ^{p}}{(x+h-h\alpha )_{h}^{(1-\alpha +\beta )}}\,d_{h}x \biggr] . \end{aligned}$$

By using (2.4), (2.5), (2.6) and (2.7) we obtain

3.14 $$\begin{aligned} \int_{a}^{\infty }\frac{ [ x_{h}^{(\beta )} ] ^{p} \,d_{h}x}{(x+h-h\alpha )_{h}^{(1-\alpha +\beta )}} \leq & \int _{a}^{\infty }\frac{(x+\beta (p-1)h)_{h}^{(p\beta )}\,d_{h}x}{(x+h-h\alpha )_{h}^{(1-\alpha +\beta )}} \\ =&\int_{a}^{\infty }\bigl(x+\beta (p-1)h\bigr)_{h}^{(\beta (p-1)+\alpha-1)}\,d_{h}x \\ =&\frac{\int_{a}^{\infty }D_{h} ( (x+\beta (p-1)h)_{h}^{(\beta (p-1)+\alpha )} )\,d_{h}x}{\beta (p-1)+\alpha } \\ =&\frac{1}{\vert \beta (p-1)+\alpha \vert }\bigl(a+\beta (p-1)h\bigr)_{h}^{(\beta (p-1)+\alpha )} \end{aligned}$$

and

3.15 $$\begin{aligned} (a-h\alpha )_{h}^{(1-\alpha +\beta )} =&(a-h\alpha )_{h}^{(-\alpha )}\bigl(a-h(p \beta +1)\bigr)_{h}^{(\beta (1-p)}a_{h}^{(p\beta +1)} \\ =&(a-h\alpha )_{h}^{(-\alpha )}\bigl(a-h(p\beta +1)\bigr)_{h}^{(\beta (1-p)} \int_{a}^{\infty }D_{h} \bigl[t_{h}^{(p\beta +1)} \bigr]\,d_{h}t \\ \leq &(a-h\alpha )_{h}^{(-\alpha )}\bigl(a-h(p\beta +1) \bigr)_{h}^{(\beta (1-p)}\vert \beta p+1 \vert \int _{a}^{\infty } \bigl[ t_{h}^{(\beta )} \bigr] ^{p}\,d _{h}t. \end{aligned}$$

According to (2.6), (3.13), (3.14) and (3.15) we can deduce that

$$ L(f_{\beta })\geq \biggl( \frac{1}{1-\alpha +\beta } \biggr) ^{p} \biggl[ \int _{0}^{\infty }f_{\beta }^{p}(x)\,d_{h}x- \theta_{\beta }(a) \int _{0}^{\infty }f_{\beta }^{p}(x)\,d_{h}x \biggr] , $$

where $\theta_{\beta }(a):= \frac{p\vert \beta p+1 \vert }{\vert \beta (p-1)+\alpha \vert }(a+\beta (p-1)h)_{h}^{( \beta (p-1))}(a-h(p\beta +1))_{h}^{(\beta (1-p))}\rightarrow 0,\quad \varepsilon \rightarrow 0$.

Therefore, $\lim_{\varepsilon \rightarrow 0}\frac{L(f_{\beta })}{\int_{0}^{\infty }f_{\beta }^{p}(x)\,d_{h}x}\geq \lim _{\varepsilon \rightarrow 0} ( \frac{1}{1-\alpha +\beta } ) ^{p}= ( \frac{p}{p-p\alpha -1} ) ^{p}$, which implies that the constant $[ \frac{p}{p-\alpha p-1} ] ^{p}$ in (3.1) in sharp.

Let $p = 1$. By using Definition 2.3 and (2.5) we get

$$\begin{aligned} \int _{0}^{\infty }x^{(\alpha -1)}_{h} \int _{0}^{\delta _{h}(x)}\frac{1}{t_{h}^{(\alpha )}} f(t)\,d_{h}t\,d_{h}x =& \sum _{i=0}^{\infty }h(ih)_{h}^{(\alpha -1)} \sum _{k=0}^{i}h(kh- \alpha h)_{h}^{(-\alpha )}f(kh) \\ =&\sum _{k=0}^{\infty }h(kh-\alpha h)_{h}^{(-\alpha )}f(kh) \sum _{i=k}^{\infty }h(ih)_{h}^{(\alpha -1)} \\ =& \int _{0}^{\infty }(t-\alpha h)_{h}^{(-\alpha )}f(t) \int _{t}^{\infty }x_{h}^{(\alpha -1)}\,d_{h}x\,d_{h}t\\ =&\frac{1}{\alpha } \int _{0}^{\infty }(t-\alpha h)_{h}^{(- \alpha )}f(t) \int _{t}^{\infty }D_{h} \bigl( x_{h}^{(\alpha )} \bigr) d _{h}x\,d_{h}t\\ =&-\frac{1}{\alpha } \int _{0}^{\infty }f(t) (t-\alpha h)_{h} ^{(-\alpha )}t_{h}^{(\alpha )}\,d_{h}t =- \frac{1}{\alpha } \int _{0}^{\infty }f(t)\,d_{h}t, \end{aligned}$$

which means that (3.1) holds even with equality in this case. The proof is complete. □

### Proof of Theorem 3.2

Let $0< p<1$. By using (2.4), (2.5) and (2.7) we get

3.16 $$\begin{aligned} \bigl[ x^{(\alpha -1)}_{h} \bigr] ^{p} =& \bigl[ x^{(\alpha -1)}_{h} \bigr] ^{p-1}x^{(\alpha -1)}_{h} \\ =& \biggl[ x^{(\alpha -\frac{1}{p'})}_{h}\biggl(x-\biggl(\alpha - \frac{1}{p'}\biggr)h\biggr)^{(- \frac{1}{p})}_{h} \biggr] ^{p-1} x^{(\alpha -\frac{1}{p'}-1)}_{h}\biggl(x+h-\biggl( \alpha - \frac{1}{p'}\biggr)h\biggr)^{(\frac{1}{p'})}_{h} \\ \geq & \bigl[ x^{(\alpha -\frac{1}{p'})}_{h} \bigr] ^{p-1}x^{(\alpha - \frac{1}{p'}-1)}_{h} \frac{(x-(\alpha -\frac{1}{p'})h)^{(\frac{1}{p'})} _{h}}{ [ (x-(\alpha -\frac{1}{p'})h)^{(-\frac{1}{p})}_{h} ] ^{1-p}} \\ \geq & \bigl[ x^{(\alpha -\frac{1}{p'})}_{h} \bigr] ^{p-1}x^{(\alpha - \frac{1}{p'}-1)}_{h} \frac{(x-(\alpha -\frac{1}{p'})h)^{(\frac{1}{p'})} _{h}}{(x-(\alpha -\frac{1}{p'})h)^{(-\frac{1-p}{p})}_{h}} \\ =& \bigl[ x^{(\alpha -\frac{1}{p'})}_{h} \bigr] ^{p-1}x_{h}^{(\alpha - \frac{1}{p'}-1)} \\ =& \biggl[ \biggl(x-\biggl(\alpha -\frac{1}{p'}\biggr)h \biggr)^{(\frac{1}{p'}-\alpha )}_{h} \biggr] ^{1-p}x_{h}^{(\alpha -\frac{1}{p'}-1)} \\ \geq & \biggl[ \frac{1}{\frac{1}{p'}-\alpha } \biggr] ^{p-1} \biggl[ \int _{0}^{\delta_{h}(x)}\biggl(t-\biggl(\alpha + \frac{1}{p}\biggr)h\biggr)^{(-\alpha -\frac{1}{p})} _{h}\,d_{h}t \biggr] ^{1-p} x_{h}^{(\alpha -\frac{1}{p'}-1)} \end{aligned}$$

and

3.17 $$\begin{aligned} \biggl[ \frac{1}{t_{h}^{(\alpha )}} \biggr] ^{p} =& \bigl[ (t- \alpha h)_{h} ^{(-\alpha )} \bigr] ^{p-1}\frac{1}{t_{h}^{(\alpha )}} \\ =& \biggl[ \biggl(t-\biggl(\alpha +\frac{1}{p}\biggr) h \biggr)_{h}^{(-\alpha -\frac{1}{p})}(t- \alpha h)_{h}^{(\frac{1}{p})} \biggr] ^{p-1}\frac{1}{t_{h}^{(\alpha - \frac{1}{p'})}(t-(\alpha -\frac{1}{p'})h)_{h}^{(\frac{1}{p'})}} \\ =& \biggl[ \biggl(t-\biggl(\alpha +\frac{1}{p}\biggr)h \biggr)_{h}^{(-\alpha -\frac{1}{p})} \biggr] ^{p-1}\frac{1}{t_{h}^{(\alpha -\frac{1}{p'})}}\frac{(t-\alpha h)_{h} ^{(-\frac{1}{p'})}}{ [ (t-\alpha h)_{h}^{(\frac{1}{p})} ] ^{1-p}} \\ \geq & \biggl[ \biggl(t-\biggl(\alpha +\frac{1}{p}\biggr)h \biggr)_{h}^{(-\alpha -\frac{1}{p})} \biggr] ^{p-1}\frac{1}{t_{h}^{(\alpha -\frac{1}{p'})}}\frac{(t-\alpha h)_{h} ^{(-\frac{1}{p'})}}{(t-\alpha h)_{h}^{(\frac{1-p}{p})}} \\ =& \biggl[ \biggl(t-\biggl(\alpha +\frac{1}{p}\biggr)h \biggr)_{h}^{(-\alpha -\frac{1}{p})} \biggr] ^{p-1}\frac{1}{t_{h}^{(\alpha -\frac{1}{p'})}}. \end{aligned}$$

Moreover, by using Definition 2.3, (3.16) and (3.17), and applying the Hölder inequality with powers $1/p$ and $1/(1-p)$, we obtain

$$\begin{aligned} \frac{L(f)}{ [ \frac{1}{\frac{1}{p'}-\alpha } ] ^{p-1}} \geq & \int _{0}^{\infty }x_{h}^{(\alpha -\frac{1}{p'}-1)} \biggl[ \int _{0}^{\delta_{h}(x)}\biggl(t-\biggl(\alpha +\frac{1}{p} \biggr)h\biggr)^{(-\alpha - \frac{1}{p})}_{h}\,d_{h}t \biggr] ^{1-p} \\ & {}\times \biggl[ \int _{0}^{\delta_{h}(x)}\frac{1}{t_{h}^{(\alpha )}} f(t)\,d_{h}t \biggr] ^{p}\,d_{h}x \\ =&\sum _{k=0}^{\infty }{h}(kh)_{h}^{(\alpha -\frac{1}{p'}-1)} \Biggl[ \sum _{i=0}^{k}h\biggl(ih-\biggl(\alpha +\frac{1}{p}\biggr)h\biggr)^{(-\alpha - \frac{1}{p})}_{h} \Biggr] ^{1-p} \Biggl[ \sum _{i=0}^{k}h \frac{f(ih)}{(ih)_{h} ^{(\alpha )}} \Biggr] ^{p} \\ \geq &\sum _{k=0}^{\infty }h(kh)_{h}^{(\alpha -\frac{1}{p'}-1)} \sum _{i=0}^{k}h \biggl[ \biggl(ih-\biggl(\alpha +\frac{1}{p}\biggr)h\biggr)^{(-\alpha - \frac{1}{p})}_{h} \biggr] ^{1-p} \biggl[ \frac{f(ih)}{(ih)_{h}^{(\alpha )}} \biggr] ^{p} \\ =& \sum _{i=0}^{\infty }{h} f^{p}(ih) \biggl[ \biggl(ih-\biggl(\alpha + \frac{1}{p}\biggr)h\biggr)^{(-\alpha -\frac{1}{p})}_{h} \biggr] ^{1-p} \biggl[ \frac{1}{(ih)_{h} ^{(\alpha )}} \biggr] ^{p}\sum_{k=i}^{\infty }h(kh)_{h}^{(\alpha -\frac{1}{p'}-1)} \\ =& \int _{0}^{\infty }f^{p}(t) \biggl[ \biggl(t-\biggl( \alpha +\frac{1}{p}\biggr)h\biggr)^{(- \alpha -\frac{1}{p})}_{h} \biggr] ^{1-p} \biggl[ \frac{1}{t_{h}^{(\alpha )}} \biggr] ^{p} \int _{t}^{\infty }x_{h}^{(\alpha -\frac{1}{p'}-1)}\,d _{h}x\,d_{h}t \\ \geq &\frac{1}{\frac{1}{p'}-\alpha } \int _{0}^{\infty }f^{p}(t) \biggl[ \biggl(t-\biggl( \alpha +\frac{1}{p}\biggr)h\biggr)^{(-\alpha -\frac{1}{p})}_{h} \biggr] ^{1-p} \biggl[ \biggl(t-\biggl(\alpha +\frac{1}{p}\biggr)h \biggr)_{h}^{(-\alpha -\frac{1}{p})} \biggr] ^{p-1} \\ &{}\times \frac{1}{t_{h}^{(\alpha -\frac{1}{p'})}} \int _{t}^{ \infty }D_{h} \bigl[ x_{h}^{(\alpha -\frac{1}{p'})} \bigr]\,d_{h}x\,d_{h}t \\ =&\frac{1}{\frac{1}{p'}-\alpha } \int _{0}^{\infty }f^{p}(t)\,d _{d}t, \end{aligned}$$

i.e.

$$\begin{aligned} \biggl[ \frac{1}{p'}-\alpha \biggr] ^{p}L(f) \geq & \int _{0}^{ \infty }f^{p}(t)\,d_{d}t. \end{aligned}$$

Therefore, we deduce that inequality (3.2) holds for all functions $f \geq 0$ and the left hand side of (3.2) is finite.

Finally, we prove that the constant $[ \frac{p-1}{p}-\alpha ] ^{p}$ in inequality (3.2) is sharp. Let $x,a\in \mathbb{T}_{0}$, be such that $a< x$, and $f_{\beta }(t)=t_{h}^{( \beta )}\chi_{[a,\infty )}(t)$, where $\alpha -1<\beta <-\frac{1}{p}$. By using (2.4), (2.5) and (2.8) we find that

$$\begin{aligned} \int _{0}^{\infty }f_{\beta }(t)\,d_{h}t =& \int _{a}^{ \infty } \bigl( t_{h}^{(\beta )} \bigr) ^{p}\,d_{h}t\leq \int _{a} ^{\infty }t_{h}^{(\beta p)}\,d_{h}t \\ =&\frac{1}{p\beta +1} \int _{a}^{\infty }D_{h} \bigl[ t_{h}^{( \beta p+1)} \bigr]\,d_{h}t \\ =&\frac{a_{h}^{(p\beta +1)}}{\vert p\beta +1 \vert }< \infty \end{aligned}$$

and

3.18 $$\begin{aligned} L(f_{\beta }) =&\sum _{i=0}^{\infty }{h} \Biggl[ (ih)^{(\alpha -1)} _{h}\sum _{k=0}^{i}{h}(kh- \alpha h)_{h}^{(-\alpha )}f_{\beta }(kh) \Biggr] ^{p} \\ =& \sum _{i=0}^{\frac{a}{h}-1}{h} \Biggl[ (ih)^{(\alpha -1)}_{h} \sum _{k=0}^{i}{h}(kh-\alpha h)_{h}^{(-\alpha )}f_{\beta }(kh) \Biggr] ^{p} \\ &{}+ \sum _{i=\frac{a}{h}}^{\infty }{h} \Biggl[ (ih)^{(\alpha -1)} _{h}\sum _{k=0}^{i}{h}(kh-\alpha h)_{h}^{(-\alpha )}f_{\beta }(kh) \Biggr] ^{p} \\ =& \int _{a}^{\infty } \biggl[ x^{(\alpha -1)}_{h} \int _{0} ^{\delta_{h}(x)}(t-\alpha h)_{h}^{(-\alpha +\beta )}\,d_{h}t \biggr] ^{p}\,d _{h}x \\ =& \biggl[ \frac{1}{1-\alpha +\beta } \biggr] ^{p} \int _{a}^{ \infty } \biggl[ x^{(\alpha -1)}_{h} \int _{0}^{\delta_{h}(x)}D_{h} \bigl[ (t-\alpha h)_{h}^{(1-\alpha +\beta )} \bigr]\,d_{h}t \biggr] ^{p}\,d _{h}x \\ \leq & \biggl[ \frac{1}{1-\alpha +\beta } \biggr] ^{p}\int _{a}^{ \infty } \bigl[ x^{(\alpha -1)}_{h}(x+h- \alpha h)_{h}^{(1-\alpha + \beta )} \bigr] ^{p}\,d_{h}x \\ =& \biggl[ \frac{1}{1-\alpha +\beta } \biggr] ^{p} \int _{a}^{ \infty } \bigl[ x_{h}^{(\beta )} \bigr] ^{p}\,d_{h}x= \biggl( \frac{1}{1- \alpha +\beta } \biggr) ^{p} \int _{0}^{\infty }f_{\beta }^{p}(x)\,d _{h}x. \end{aligned}$$

From (3.18) its follows that

$$ \sup _{\alpha -1\geq \beta \geq -\frac{1}{p}}\frac{\int _{0}^{\infty }f_{\beta }(t)\,d_{h}t}{L(f_{\beta })}=\sup _{\alpha -1< \beta < -\frac{1}{p}} [ 1-\alpha +\beta ] ^{p}= \biggl[ \frac{1}{p'}-\alpha \biggr] ^{p}, $$

and this shows that the constant $[ \frac{p-1}{p}-\alpha ] ^{p}$ in inequality (3.2) is sharp. The proof is complete. □

Now, let us comment which discrete analogue of Hardy inequality we are getting from the Hardy *h*-inequality. Directly from the proof of Theorems 3.1 and 3.2 we obtain the following discrete inequality, which is of independent interest.

### Remark 3.4

On the basis of Definitions 2.4–2.5 we get

$$\begin{aligned} \sum _{n=0}^{\infty } \Biggl[ \frac{\Gamma ( \frac{nh}{h}+1 ) }{ \Gamma ( \frac{nh}{h}+2-\alpha ) } \sum_{k=0}^{n}\frac{ \Gamma ( \frac{kh}{h}+1-\alpha ) }{\Gamma ( \frac{nh}{h}+1 ) }a _{k} \Biggr] ^{p}\leq \biggl( \frac{p}{p-\alpha p-1} \biggr) ^{p} \sum_{n=0}^{\infty }a^{p}_{k}, \quad a_{k}\geq 0, \end{aligned}$$

for $p\geq 1$ and $\alpha < 1-1/p$.
